# Infrared Multiphoton Dissociation Enables Top-Down Characterization of Membrane Protein Complexes and G Protein-Coupled Receptors

**DOI:** 10.1002/anie.202305694

**Published:** 2023-07-25

**Authors:** Corinne A. Lutomski, Tarick J. El-Baba, Joshua D. Hinkle, Idlir Liko, Jack L. Bennett, Neha V. Kalmankar, Andrew Dolan, Carla Kirschbaum, Kim Greis, Leonhard H. Urner, Parth Kapoor, Hsin-Yung Yen, Kevin Pagel, Christopher Mullen, John E. P. Syka, Carol V. Robinson

**Affiliations:** Physical and Theoretical Chemistry Laboratory, Department of Chemistry, University of Oxford, United Kingdom; Kavli Institute for Nanoscience Discovery, Dorothy Crowfoot Hodgkin Building, University of Oxford, Oxford, United Kingdom; Physical and Theoretical Chemistry Laboratory, Department of Chemistry, University of Oxford, United Kingdom; Kavli Institute for Nanoscience Discovery, Dorothy Crowfoot Hodgkin Building, University of Oxford, Oxford, United Kingdom; Thermo Fisher Scientific, San Jose, California, United States of America; OMass Therapeutics, Oxford, United Kingdom; Physical and Theoretical Chemistry Laboratory, Department of Chemistry, University of Oxford, United Kingdom; Kavli Institute for Nanoscience Discovery, Dorothy Crowfoot Hodgkin Building, University of Oxford, Oxford, United Kingdom; Physical and Theoretical Chemistry Laboratory, Department of Chemistry, University of Oxford, United Kingdom; Kavli Institute for Nanoscience Discovery, Dorothy Crowfoot Hodgkin Building, University of Oxford Oxford, United Kingdom; Physical and Theoretical Chemistry Laboratory, Department of Chemistry, University of Oxford, United Kingdom; Kavli Institute for Nanoscience Discovery, Dorothy Crowfoot Hodgkin Building, University of Oxford Oxford, United Kingdom; Institute of Chemistry and Biochemistry, Freie Universität Berlin, Berlin, Germany; Fritz Haber Institute of the Max Planck Society, Berlin, Germany; Institute of Chemistry and Biochemistry, Freie Universität Berlin, Berlin, Germany; Fritz Haber Institute of the Max Planck Society Berlin, Germany; Institute of Chemistry and Biochemistry, Freie Universität Berlin Berlin, Germany; Fritz Haber Institute of the Max Planck Society Berlin, Germany; Department of Chemistry and Chemical Biology TU Dortmund University Dortmund, Germany; OMass Therapeutics, Oxford, United Kingdom; OMass Therapeutics Oxford, United Kingdom; Institute of Biological Chemistry Academia Sinica Taipei, Taiwan; Institute of Chemistry and Biochemistry Freie Universität Berlin Berlin, Germany; Fritz Haber Institute of the Max Planck Society Berlin, Germany; Thermo Fisher Scientific, San Jose, California, United States of America; Thermo Fisher Scientific, San Jose, California, United States of America; Physical and Theoretical Chemistry Laboratory, Department of Chemistry, University of Oxford, United Kingdom; Kavli Institute for Nanoscience Discovery, Dorothy Crowfoot Hodgkin Building, University of Oxford, Oxford, United Kingdom

**Keywords:** Mass Spectrometry, Membrane Proteins, Infrared Multiphoton Dissociation, Tandem Mass Spectrometry

## Abstract

Membrane proteins are challenging to analyze by native mass spectrometry (MS) as their hydrophobic nature typically requires detergent micelles that are removed prior to analysis via collisional activation. There is however a practical limit to the amount of energy which can be applied, which often precludes subsequent characterization by top-down MS. To overcome this barrier, we have applied a modified Orbitrap Eclipse Tribrid mass spectrometer coupled to an infrared laser within a high-pressure linear ion trap. We show how tuning the intensity and time of incident photons enables liberation of membrane proteins from detergent micelles. Specifically, we relate the ease of micelle removal to the infrared absorption of detergents in both condensed and gas phases. Top-down MS via infrared multiphoton dissociation (IRMPD), results in good sequence coverage enabling unambiguous identification of membrane proteins and their complexes. By contrasting and comparing the fragmentation patterns of the ammonia channel with two class A GPCRs, we identify successive cleavage of adjacent amino acids within transmembrane domains. Using gas-phase molecular dynamics simulations, we show that areas prone to fragmentation maintain aspects of protein structure at increasing temperatures. Altogether, we propose a rationale to explain why and where in the protein fragment ions are generated.

## Introduction

Native mass spectrometry (MS) is a well-established tool in structural biology.^[1]^ The use of nondenaturing buffers during electrospray ionization has allowed for the preservation of noncovalent interactions between multi-component protein complexes, as well as their ligands, cofactors, or other bound proteins.^[2]^ It represents the gold-standard for understanding the interplay between interaction and function, as binding events result in changes in mass that can be followed and dissected within the mass spectrometer.^[3]^ Membrane proteins, however, present unique challenges in native MS due to their hydrophobic nature which makes them insoluble and prone to aggregation in traditional native MS buffers. While it is now possible to carry out native mass measurements directly from endogenous membranes,^[4]^ the current standard for targeted analysis of membrane proteins still requires stabilization of the exposed hydrophobic regions with membrane mimetics such as detergents, nanodiscs, and amphipols, among others.^[5]^ Detergents remain the most widely used membrane mimetic, due to the ability to readily liberate intact membrane protein complexes from micelles in the gas phase.

In native MS, the detergent micelles that envelop the membrane protein must be stripped before mass-to-charge (*m/z*) analysis, typically using collisional activation (e.g. ion acceleration into an inert gas) within dedicated regions in the mass spectrometer. Detergents that are considered most compatible with native MS, such as glycol ethers like tetraethylene glycol monooctyl ether (C_8_E_4_), require low levels of collisional activation in order to generate well-resolved mass spectra of membrane proteins.^[5a]^ Despite this, they are considered harsh delipidating agents ^[6]^ and have been shown to distort preferential lipid interactions ^[7]^ as well as protein-protein interactions.^[8]^ In contrast, non-ionic maltoside detergents, such as dodecyl-*n*-maltopyranoside (DDM), are minimally disruptive to non-covalent interactions, such as protein-lipid interactions, and are widely used in structural biology and mass spectrometry. However, as there is a practical limit to the amount of collisional activation that can be imparted in the mass spectrometer, DDM is difficult to remove and often results in heterogeneous mass spectra where detergent remains bound to the membrane protein, even at the maximum energy input available on commercial instrumentation. Alas, many mammalian membrane proteins, such as G protein-coupled receptors (GPCRs), typically require solubilization in detergents such as DDM.^[9]^ To overcome this, we previously synthesized detergents tailored for native MS, where chemical moieties from several common detergents were combined.^[7, 10]^ These designer detergents were found to be mildly delipidating, were applicable to many types of membrane proteins, including GPCRs,^[10a]^ and importantly, were comparatively easier to remove in the mass spectrometer. Still, even with these designer detergents in the native MS toolbox, ^[11]^ tailoring the selection of a non-denaturing detergent that is minimally disruptive to non-covalent interactions *and* can be effectively removed in the MS instrument is still a laborious and unpredictable process.

Despite the challenges associated with removing detergent micelles in the gas phase, there are many case studies in which membrane protein complexes have been successfully liberated from detergent micelles and/or mimetics prior to mass analysis, enabling and the elucidation of complex stoichiometry and the intact masses of bound lipids.^[12]^ Recently, the precise molecular identification of lipids and small molecules liberated from native membrane protein complexes was enabled using multi-stage tandem mass spectrometry (MS^n^).^[13]^ Topdown sequencing of native membrane proteins, however, remains challenging due to their high molecular weight, low charge density, and the practical limits to the amount of activation energy that can be imparted in commercial mass spectrometers.

Collision-based modalities are some of the most commonly used fragmentation methods and are available on most instruments; top-down sequencing by collisional activation has been demonstrated for a number of membrane proteins.^[14]^ However, the sequence information obtained is limited by difficulties associated with converting kinetic energy into internal energy via collisions, which is inversely proportional to the mass of the analyte ion.^[15]^ Furthermore, under native MS conditions, bond cleavage is also impacted by the strength of noncovalent interactions (e.g. protein-protein, protein-detergent, and within detergent micelles).^[14b]^ Thus, collision-based strategies are more effective for soluble protein complexes than for their membrane embedded counterparts.

Instead, photon-based methods such as ultraviolet photodissociation (UVPD)^[16]^ and infrared multiphoton dissociation (IRMPD)^[17]^ have proven advantageous for top-down MS of soluble proteins and their complexes. Meanwhile, the use of photon-based modalities for native top-down of membrane proteins is still emerging.^[14c]^ In principle, ions in the path of the laser beam are continuously driven to vibrationally excited states. Therefore, in photon-based methods, the energy deposition is typically agnostic to the charge state and molecular weight of the precursor ion. However, both properties influence the conversion of input energy into peptide bond dissociation. Previously reported top-down fragmentation of the 79 kDa mechanosensitive channel of large conductance (MscL) via UVPD resulted in an impressive 54% sequence coverage directly from the pentameric complex.^[14c]^ However, for the ammonia channel (AmtB) ^[14c]^ a 127 kDa homotrimer, fragmentation via two different modalities, collisional activation and UVPD, achieved <20% sequence coverage. Indeed, the comparatively lower sequence coverage is a consequence of the large molecular weight as well as the increased number of noncovalent interactions in the trimeric complex. In spite of these tools which enable native top-down MS,^[14c, 16, 18]^ its widespread use in membrane protein characterization is still underdeveloped. It demands that protein sequence ions are generated robustly from high molecular weight proteins with low charge density, which is further complicated by the fact that membrane proteins are embedded in heterogeneous membranes or mimetics. While IRMPD was used previously to liberate membrane proteins from detergents ^[12e, 19]^, the use of IRMPD to sequence native membrane proteins remains relatively unexplored.

We envisioned an experiment where IR activation would enable efficient removal of detergent micelles from protein complexes, followed by subsequent top-down fragmentation of native membrane proteins by IRMPD in the same experiment. Here, we utilize a modified Orbitrap Eclipse Tribrid MS, interfaced with a continuous wave far infrared (IR) CO_2_ laser such that the beam waist is focused into the high-pressure cell of the dual cell quadrupole linear ion trap (QLIT).^[20]^ We find that tuning the intensity and time of incident photons enables efficient and selective removal of detergents from membrane proteins prior to mass analysis. To understand its potential for this application, we studied the gas- and condensed-phase IR spectra to probe the photochemical properties of standard MS detergents. As the QLIT enables MS^n^ analysis, we can selectively isolate and employ IRMPD to induce fragmentation of membrane protein complexes and GPCRs liberated from ‘difficult’ detergents. Large, multiply charged fragments are produced, even from trimeric complexes >127 kDa, yielding fragments corresponding to cleavage at consecutive peptide bonds, enabling identification of short sequences of amino acids (i.e. sequence tags) for unambiguous protein identification. Identified stretches of amino acids originate almost exclusively from cleavages within helical transmembrane regions. Their localization prompted the consideration of possible mechanisms which result in both sequence and domain-derived fragmentation patterns. We used gas-phase molecular dynamics (MD) simulations to interrogate the relationship between structure and fragmentation and present a framework with three representative membrane proteins to establish how fragmentions are generated, and why they arise. Overall, we achieve sufficient sequence coverage (>20%) to enable unambiguous identification of membrane proteins and their complexes.

## Results and Discussion

### Liberation of Membrane Proteins from Detergent Micelles via Infrared Irradiation

**Figure 1:**
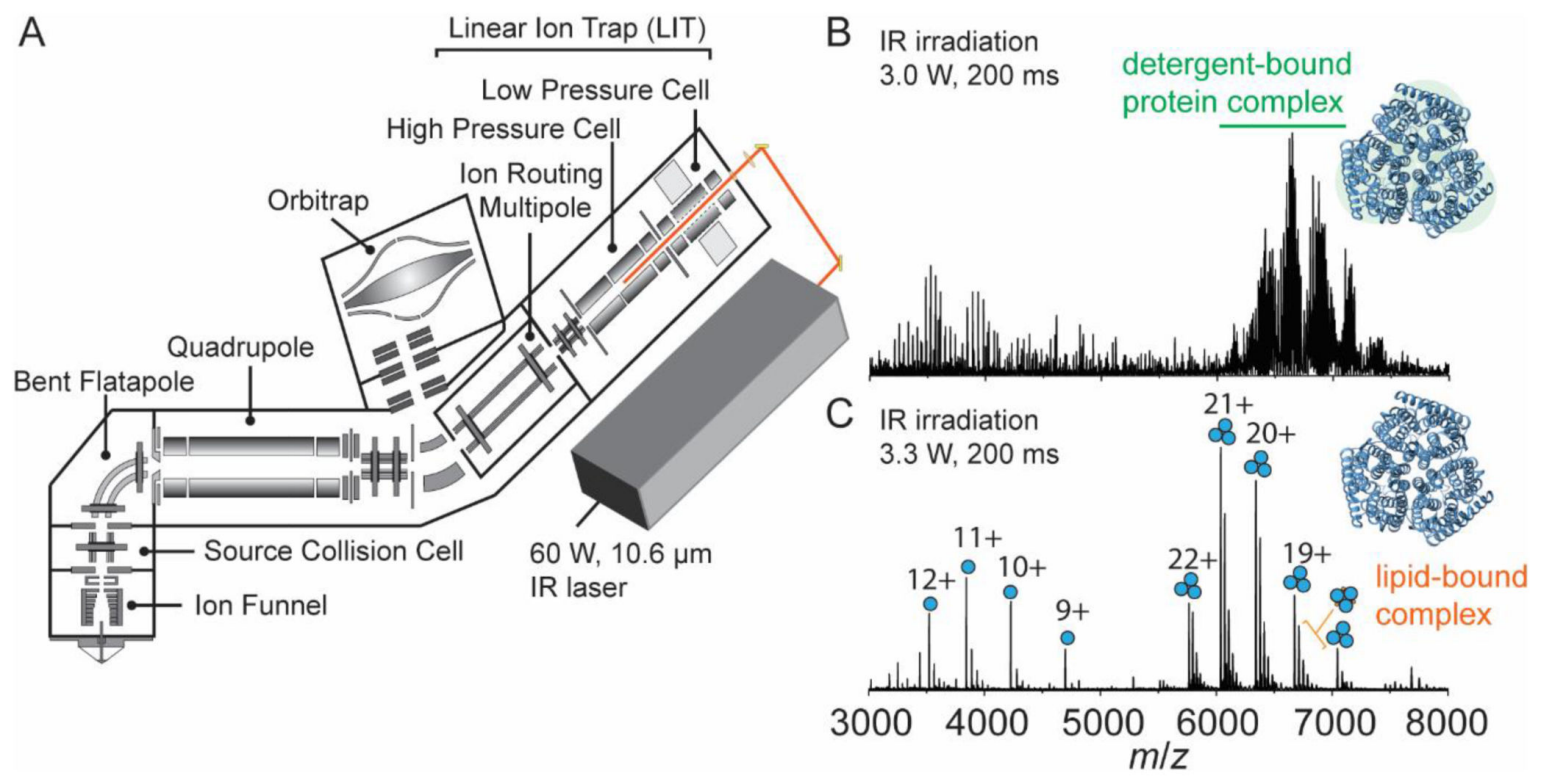
Overview of the modified instrument for membrane protein native MS. (A) Schematic of the modified Orbitrap Eclipse Tribrid which includes an infrared laser directed into the high-pressure cell of the quadrupole linear ion trap (QLIT). Ionized proteomicelles are transferred into the high-pressure QLIT where entire population of ions are subjected to irradiation with IR photons before being transferred into the Orbitrap for mass analysis. Laser output power (W) and irradiation time (ms) can be tuned to fully liberate membrane proteins from the detergent micelle. (B) a native mass spectrum of the trimeric ammonia channel (AmtB) at 3.0 W of output power and 200 ms irradiation time. The peaks corresponding to trimeric AmtB remain largely detergent-bound. (C) a native mass spectrum of AmtB at 3.3 W output power and 200 ms irradiation time. Well-resolved charge states corresponding to the intact AmtB trimer with up to five bound phospholipids are observed.

We previously described an instrument capable of identifying the molecular composition of small molecule ligands and lipids bound to membrane protein complexes using multistage tandem mass spectrometry (MS^n^) on an Orbitrap Eclipse.^[13]^ Here, we describe a further modification in which we interface a continuous wave IR CO_2_ laser to the back of the QLIT manifold, allowing for 943 cm^-1^ (10.6 μm) photons to be incident onto ions stored in the high-pressure cell of the QLIT (Fig. 1A). In this arrangement, ionized proteomicelles, generated by nanoelectrospray ionization, are transferred to the high-pressure cell. The entire population of ions is then subjected to irradiation with IR photons prior to transfer to the Orbitrap for detection or *m/z* isolation and subsequent fragmentation. Utilizing the IR-based approach to remove detergent micelles offers control over two tunable parameters: laser output power (up to 60 Watts) and irradiation time (milliseconds to seconds). Therefore, we obtain greater control over the liberation of membrane proteins from proteomicelles, ensuring the preservation of native complexes while fully removing membrane mimetics from the complex. We initiated our study by benchmarking the utility of the IR laser for ejecting the intact trimeric ammonia channel AmtB from DDM micelles (Fig. 1B). Low laser output power (3.0 W) at an irradiation time of 200 ms produces a poorly resolved charge state series consisting primarily of detergent-bound protein ions with m/z between 6000 – 8000. Increasing the laser output power to ~3.3 W produced two well-resolved charge state series, the most abundant of which centered at the 20^+^ charge state near m/z ~6500, corresponding to the intact trimer with a mass of 126,794 ± 1 Da, which matches well with the theoretical mass of 126,791 Da. Adjacent to the main charge state series are peaks corresponding to mass increments of ~700 – 730 Da, which are signatures of phospholipid binding (Fig. 1C). Up to five bound phospholipids can be clearly observed (Fig. S1) and have been assigned previously as highly stabilizing phosphatidylglycerols.^[21]^ The other series, around the 11+ charge state at *m/z* ~ 3700, corresponds to AmtB monomers. The satellite features adjacent to the monomer peaks are assigned as integer multiples of DDM detergent molecules bound to the monomer. Amid this distribution of peaks, phospholipids can be observed still associated with the monomeric protein, albeit at a low abundance.

Preservation of non-covalent interactions between proteins and their effectors (e.g., drugs, lipids, or other proteins) is a defining feature of native MS. To assess the ability to maintain non-covalent interactions, we systematically surveyed combinations of IR laser output power and irradiation times (Fig. S2) to determine optimal micelle-removal conditions. At low laser output power (~3.6 W) and low activation times (10 ms), we detected primarily detergent-bound protein ions. A slight increase in laser output power to ~4.5 W led to a well-resolved charge state distribution of trimers (*m*/*z* ~6500, z_avg_ ~ 20^+^). Under these conditions, we also observed several satellite peaks corresponding to lipid-bound states. Indeed, higher laser output power (~5.4 W) caused the complex to dissociate into subunits. This phenomenon has been observed in previous studies which have attributed the emergence of monomeric subunits to concurrent gas-phase unfolding and dissociation.^[22]^ Expectedly, longer irradiation times reduce the threshold for the IR laser power required to efficiently remove micelles from trimers (3.6 W for 200 ms versus 5.4 W for 10 ms). Thus, we find that the IR parameters for ejecting membrane protein ions from detergent micelles are highly tunable, and careful optimization can lead to settings that both maximize detergent removal and preserve membrane protein-ligand interactions.

**Figure 2:**
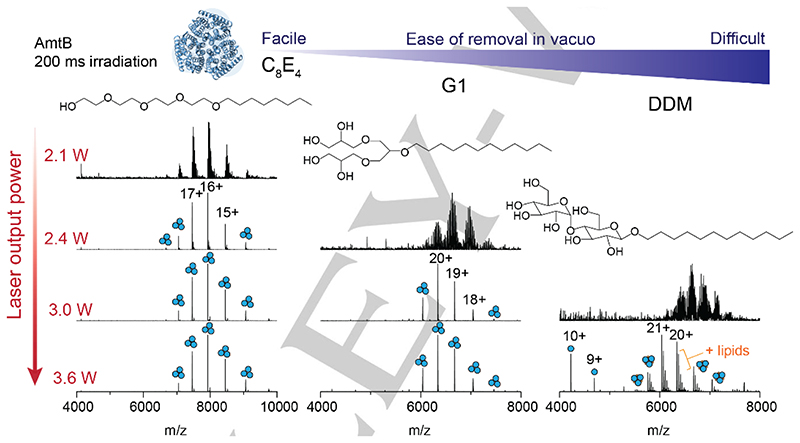
Removal of detergents from membrane protein ions by IR irradiation is highly tunable. The effect of increasing laser output power on appearance of peaks for AmtB trimers for three commonly used MS compatible detergents (C_8_E_4_, G1, and DDM). The irradiation time was fixed at 200 ms and laser output powers of 2.1,2.4, 3.0, and 3.6 W were tested. Detergents are displayed in order of ease of removal *in vacuo*, as determined by the amount of laser output power required to fully liberate membrane protein complexes resulting in well-resolved charge state peaks in the *m/z* spectra.

We next investigated the laser output power required to effect the removal of a range of native MS-compatible detergents from trimeric AmtB *in vacuo*.^[10b, 23]^ The detergents were compared based on their ease of removal *in vacuo*, which was defined previously as the ability to resolve protein peaks in the mass spectrum as a function of imparted activation energy,^[5a]^ and here, described as a function of laser output power. We first considered a glycol ether (C_8_E_4_), which typically requires low levels of collision energy, a synthetic, first-generation, dendritic oligoglycerol detergent (G1), and the non-ionic maltoside (DDM), known to require high levels of collision energy (Fig. 2). At a fixed irradiation time of 200 ms and at low laser output power (2.1 W), we could readily discern charge state series for the intact trimer embedded in C_8_E_4_, centered around the 16+ charge state at m/z ~7925, albeit with high levels of detergent adducts. An increase in laser output power to 2.4 W generated a well-resolved charge state series. Similarly, with G1 detergent, the *m/z* peak series corresponding to AmtB trimers displayed adduct peaks when irradiated with a laser output power of 2.4 W that were absent when irradiated with 3.0 W. By contrast, for DDM, a poorly resolved series of charge states for the trimer were observed following irradiation with an output power of 3.0 W; 3.6 W led a well resolved series centered at a 21+ charge state (*m/z* ~6038) with satellite peaks consistent with bound phospholipids. A peak series corresponding to monomers of AmtB was also observed at *m/z* <5000. In summary, IR activation is an efficient means of liberating membrane proteins from commonly used detergents and produces mass spectra that are comparable to collision-based micelle removal. Similar to collision-based micelle removal, different detergents will require optimization of the laser output power and irradiation time to yield comparable spectra.

Across the three different detergents tested, C_8_E_4_ required the least IR laser output power (2.4 W) to generate well-resolved charge states. G1 and DDM required a minimum output power of 3.0 and 3.6 W respectively, to obtain comparable spectra. These trends follow those previously observed for collision-based micelle removal.^[5a]^ All together, we find that laser output powers <3.6 W are ideal for liberating membrane proteins from detergent micelles while still preserving non-covalent interactions. This represents a relatively low level of activation energy as it remains well-below the energy inputs typically required to induce bond cleavage along the protein backbone by IRMPD (discussed below).^[24]^ While there are many confounding factors which influence the stability of a membrane protein in a micelle, and therefore the ease of detergent removal *in vacuo*,^[5a, 6, 10a, 14b]^ the relatively abrupt transition between detergent adducted and liberated membrane protein ions, between 3.0 W and 3.3 W was unexpected. The laser energy that is deposited into the proteomicelle is potentially withdrawn via collisional cooling with the helium gas which fills the trap at a pressure of 6 mTorr. We surmise that, if the mechanism were solely dependent on bulk heating through translation of laser energy into vibrational modes, we would observe a gradual resolution of protein peaks in the mass spectrum with increasing laser output power. These considerations prompted us to consider additional factors which may make photon-based micelle removal more effective over traditional collision-based approaches.

**Figure 3:**
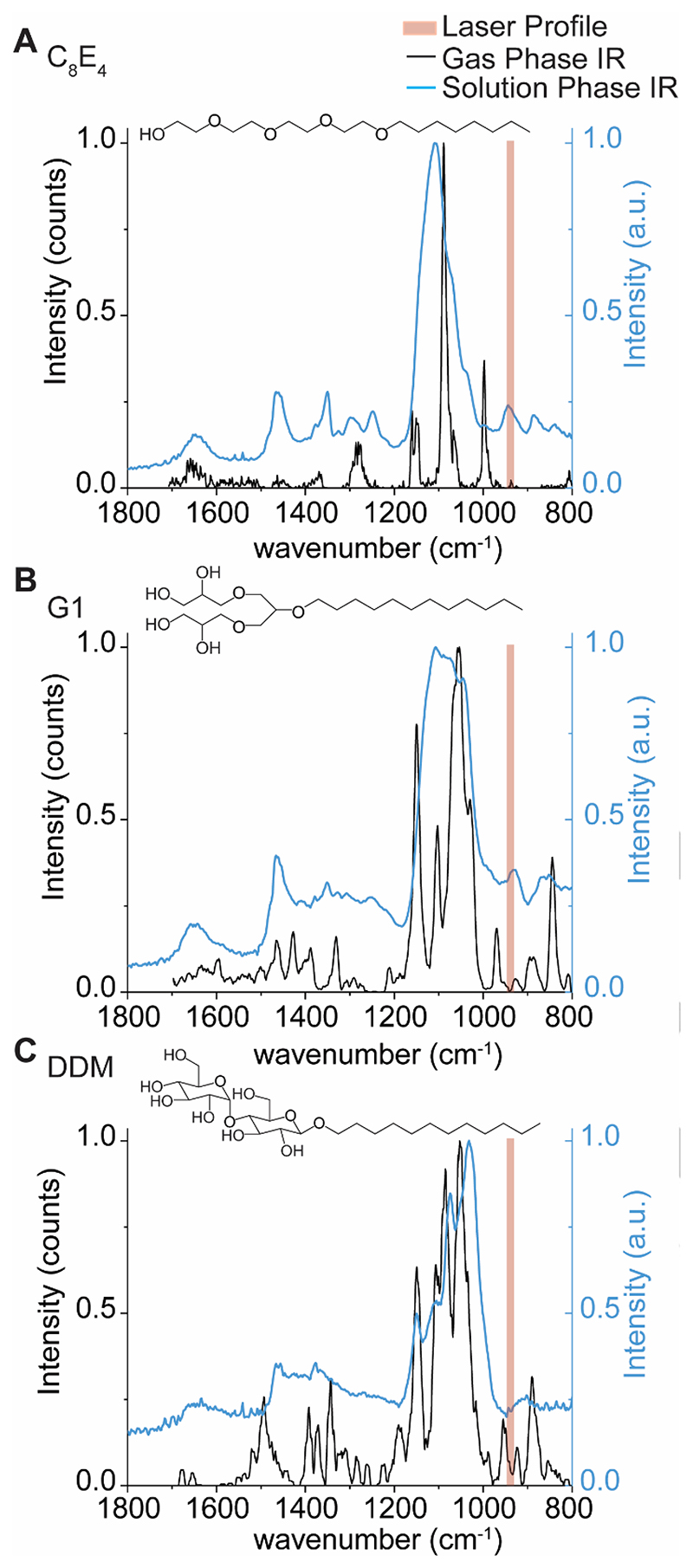
Gas- and condensed-phase IR spectra of the detergents used in the study. Gas-phase action spectra of protonated detergent ions (black trace), and condensed-phase IR spectra of self-assembled micelles in buffer (blue trace) were recorded for (A) C_8_E_4_, (B) G1, and (C) DDM to evaluate the extent to which oscillators in each detergent overlap with the anticipated profile of the IR beam. The IR beam is represented by shaded red rectangles which represent the manufacturer’s expected line width of 5.3 μm corresponding to lower and upper bounds of 936 to 947 cm^-1^, respectively.

We explored the hypothesis that IR photons are preferentially absorbed by chemical moieties in the detergent molecules/micelles. It is well-established that in CO_2_-driven IRMPD, good absorption occurs for CH_3_ rocking, O-H bending, as well as P-O, P-O-C, and P-O-P stretching.^[25]^ As the detergents described here carry O-H moieties, we hypothesized the detergents could carry distinct vibrational signatures which are on-resonant with the IR laser used here. This would allow us to affect the preferential activation of detergent. To test this hypothesis, we recorded gas-phase IR spectra of protonated detergent ions using cryogenic IR action spectroscopy (Fig. S3). Ionized detergents were captured in superfluid helium droplets, which constitute an IR-transparent, cryogenic spectroscopic matrix.^[26]^ Upon absorption of multiple IR photons, the intact ions are released from the droplets and detected by time-of-flight mass spectrometry. The appearance of released ions is then plotted as a function of wave number to yield a vibrational spectrum. For C_8_E_4_, six sharp bands were observed in the vibrational spectra (Fig. 3). Abundant bands near ~1200, 1100, and ~1000 cm^-1^ are consistent with C-O single bond stretching, which are predominant in the chemical structure of C_8_E_4_.

The gas-phase IR spectra for G1 and DDM contain additional features not as apparent in C_8_E_4_; new peaks emerge near 940 cm^-1^ that are proximal to the anticipated profile of the IR beam (943 cm^-1^, ~5.3 μm linewidth) (Fig. 3). While the gasphase spectra here illustrate the presence of absorption features in certain regions, we note that vibrational modes are strongly coupled and can be affected by anharmonic shifts ranging hundreds of wavenumbers.^[27]^ For protonated detergent ions, hydrogen bonding networks and proton sharing within oxygen-rich detergents can alter the observed vibrational frequencies. To demonstrate this, we computed the harmonic vibrations of the headgroup of protonated C_8_E_4_ using density functional theory (DFT) calculations. The computed harmonic IR spectrum (Fig. S4) reveals 21 bands between 800 and 1800 cm^-1^, and most notably, a high intensity band at 945 cm^-1^ was observed. Indeed, the harmonic at 945 cm^-1^ results from the coupled contributions of O-H bending, C-C stretching, C-H bending, and C-O stretching (Fig. S4 and Supplementary Movie S1).

We also considered this effect would be greater for detergent micelles which envelop the proteins; we hypothesized that new features in the IR spectra would also emerge upon selfassembly. We therefore sought to draw qualitative comparisons between the presence of oscillators in assembled vs monomeric detergent molecules. As this is technically challenging to achieve by gas-phase action spectroscopy, because of the effects described above, we opted to record solution phase IR spectra for C_8_E_4_, G1, and DDM (Fig. 3 and Fig S5.). We found that the solution phase spectrum for C_8_E_4_ micelles contains a prominent band which overlaps with the laser profile. This observation suggests that the multitude of hydrogen bonds in the micelle result in a stronger oscillator that overlaps with the profile of the beam. By comparison, the equivalent band is less intense, or absent, in the solution phase IR spectra of G1 and DDM, the major and minor absorption bands are offset from the profile of the laser beam (Fig. 3). The differences between the detergent micelles highlight the effect of hydrogen bonding networks on observed vibrational signatures. Adding to this, within the mass spectrometer, where there is pumping of energy during IR irradiation, further broadening of the vibrational modes occurs. Overall therefore we anticipate some spectral overlap of various oscillators with the IR laser in our experiments, than predicted from gas phase IR spectra.

Taken together, the oscillators that emerge upon detergent self-assembly generate vibrational signatures that can be targeted by IR photons with a wavenumber of ~943 cm^-1^. The presence of strong vibrational signatures on-resonant with the laser profile allow us to rationalize the low energy deposition required to liberate membrane proteins from each the detergents described above. While protein ions also contain IR-active moieties, they are poor absorbers of IR photons *in vacuo*.^[25, 28]^ Therefore, we find it likely that the detergent clusters that envelop the membrane proteins are the principal absorbers of the IR photons. It is also possible that vibrationally-excited protein ions transfer energy to the weakly bound detergents during irradiation as a means of “evaporative cooling”. But in any case, we find the low and short doses of 943 cm^-1^ photons deposit sufficient vibrational energy to dissociate the micelles that surround the membrane protein ions, without disrupting the non-covalent interactions between protein subunits and bound lipids. Thus, we find this to be a particularly useful means for liberating membrane protein ions from detergent micelles *in vacuo*.

### Infrared Multiphoton Dissociation of Membrane Proteins

**Figure 4:**
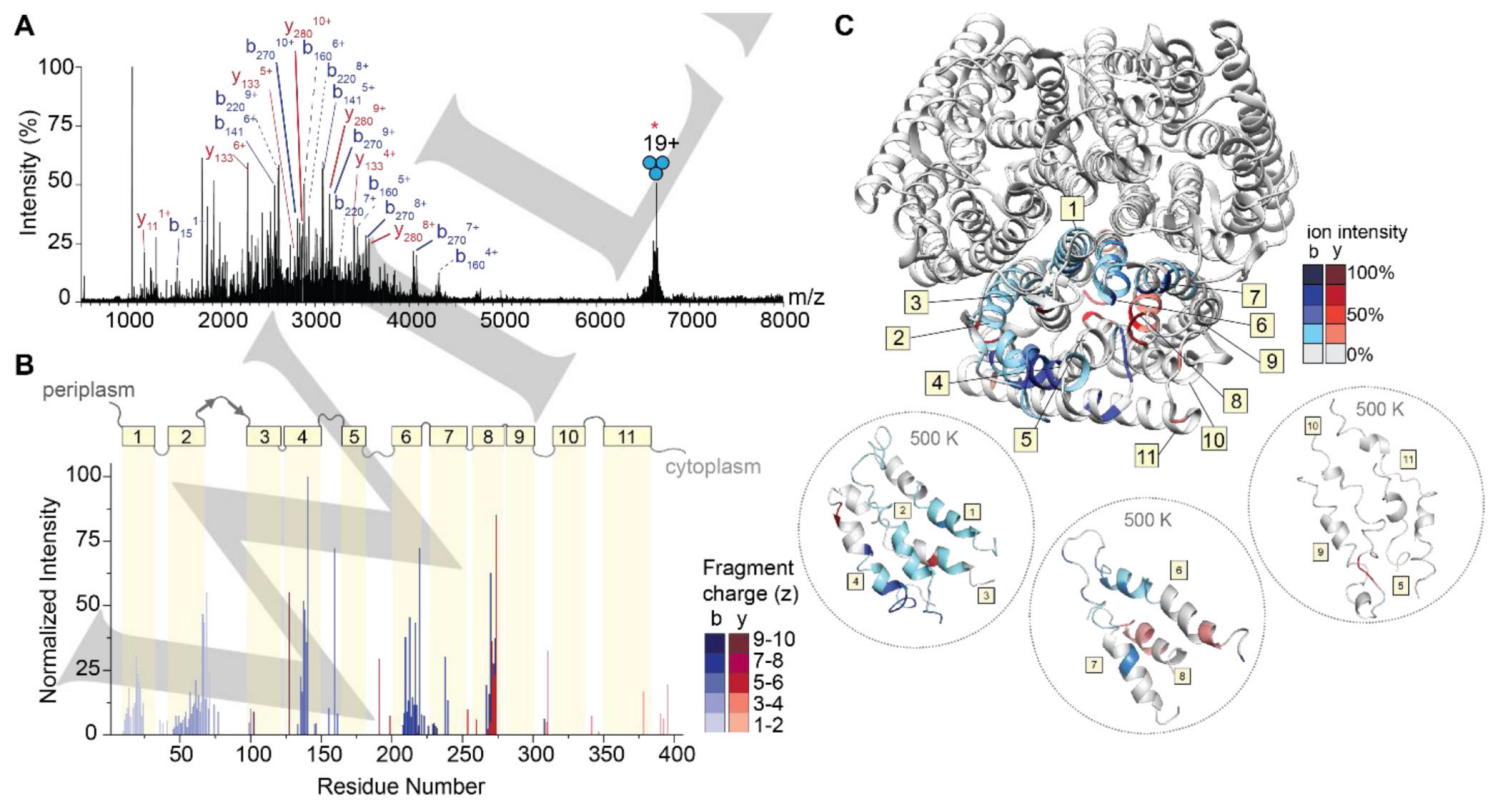
IRMPD of trimeric AmtB. (A) MS^2^ spectrum of fragment ions observed after IRMPD following ion trap isolation of the 19+ charge state at m/z 6674. Multiply charged fragments are highlighted; repeat fragments originating from like sites are grouped by dashed lines. Many assigned ions were not annotated for clarity; the remaining assignments can be found in the supporting information in Figure S6. (B) Bar plot of fragment abundance relative to the origin of cleavage (residue number), where the abundance represents the sum of normalized intensities of fragments originating from each site. The color intensity of the bar represents the weighted average charge of each assigned fragment. The topological domains of AmtB are overlaid onto the bar plot; α-helical transmembrane regions are represented by yellow boxes and numbered 1 through 11. The transmembrane regions are connected by periplasmic and cytosolic loops, represented by gray lines. (C) Sites of backbone cleavage are overlaid onto the structure of AmtB (PDB: 1U7G). Areas shaded in blue and red represent b- and y-type ions, respectively. The color intensity corresponds to the abundance of the assigned fragment. Snapshots of transmembrane helices at elevated temperature (500 K) from gas-phase molecular dynamics simulations are enclosed in dashed circles.

The gentle removal of detergent micelles by absorption of IR photons results in liberated intact protein ions that are then amenable to tandem MS experiments. To generate sequence-informative ions of a high molecular weight protein, we isolated the 19+ charge state of trimeric AmtB at m/z 6674 and subjected the ions to short irradiation (5 ms) with high laser output power (~9 W). The region between m/z 1750 and 4000 in the MS^2^ spectrum is densely populated with highly charged fragment ions (Fig. 4A and Fig. S6). Interestingly, we do not observe ejected monomers in the MS^2^ spectrum, suggesting that covalent fragmentation occurs faster than protein unfolding and subunit ejection. This effect has also been observed for a range of soluble and membrane protein complexes, where the direct fragmentation of complexes has been shown to reveal fragment ions which report on higher-order structure.^[14d]^ Many fragment ions could be assigned as b-type or y-type ions resulting from a single backbone cleavage and, as a result, 26% sequence coverage was obtained (Fig. S7). Some of the assigned ions correspond to repeat fragments, as in, the fragment ions originate from the same cleavage site but are detected as having multiple charge states. This is notable, as we often do not observe the same number of charge states for cleavages at adjacent sites, suggesting that there may be preferred sites of fragmentation (Fig. S8).

To investigate these preferred cleavage sites, the fragment ion intensities were normalized by charge state ^[29]^ allowing us to evaluate the propensity for cleavage at preferred sites based on adjacent amino acid pairs (Fig. S9). We observe high intensity sites of fragmentation resulting from traditional charge-remote X|P and D|X cleavages, namely T|P and I|P, which are known high propensity fragmentation sites for low-charge density precursors (i.e. proteins under native conditions).^[30]^ The remaining amino acid pairs resulting in the most intense fragments occur at A|G, F|G, and V|G, suggesting that there is an increased preference for fragmentation N-terminal to glycine. Despite the identification of these fragmentation “hotspots”, a correlation plot of cleavage with respect to amino acid pairs demonstrates that correlating high intensity fragment ions to sites of cleavage cannot be predicted by amino acid content alone (Fig. S10). In addition, there is little correlation with previously observed fragmentation of soluble proteins via charge remote pathways under native conditions.^[30–31]^ This observation prompted us to explore the location of observed fragmentation relative to the regions of secondary structure within the protein sequence.

To explore this, we constructed a plot of the relative abundance of each fragment with respect to the point of dissociation along the amino acid sequence of the protein (Fig. 4B). Fragment ions with multiple charge states were summed after normalizing their intensities by charge as described above. Overlaid on the bar plot is the architecture of secondary structures of AmtB, which allowed us to determine correlations between regions of fragmentation, secondary structure, and amino acid position. Most of the secondary structure of AmtB is α-helical (11 α-helices), which are connected by five ordered periplasm loops and five unstructured cytosolic loops. The N-terminus is periplasmic, and the C-terminus is in the cytosol. Focusing first on the regions of the protein which reside outside of the membrane, few associated fragment ions were observed. Basic residues in these regions, particularly arginine, are expected to sequester the already sparse mobile protons, leading to charge-remote dissociation pathways cleaving primarily C-terminal to acidic residues or N-terminal to prolines. Indeed, these fragments in the periplasmic and cytoplasmic regions arise from charge-remote fragmentation (e.g., at residues 160, 191, and 310), which are C-terminal to Asp (D|X) and Glu (E|X), as well as N-terminal to Pro (X|P), respectively. These observations are largely consistent with previous fragmentation studies of natively folded proteins.^[31]^

Turning our attention to the fragment ions that originate from bond cleavage in the membrane-spanning α-helices, we found a high propensity of successive cleavage of adjacent amino acids to enable the assignment of sequence tags. In addition, we found that certain helices fragmented with higher propensity than others. High intensity fragments originate from helices 4, 6 and 8, while we observe little to no fragmentation originating from helices 3, 5, 9, 10, and 11 (Fig. 4B). This atypical fragmentation pattern, where some transmembrane helices are “skipped,” led us to consider further the sites of fragmentation as well as their potential origin. The highest intensity fragment in the transmembrane regions corresponds to cleavage at residue 273 (helix 8) which corresponds to cleavage N-terminal to proline (T|P). Expectedly, this site of cleavage is comprised of many repeat fragments, with four unique fragments of varying charge states identified (Fig. S8). The fragments with even greater repetition (i.e. five unique charge states) occurred at residues 217 (A|G) and 220 (A|G) in helix 6. The remaining high intensity fragments originating from helix 6 occur at residues 210 (I|G), 213 (F|G). In addition, we observe complementary b-type and y-type fragments in helix 8, with the most intense y-type fragment originating from cleavage at position 270 (V|G). From these data, it is tempting to speculate that fragmentation occurring N-terminal to glycine must be a preferred cleavage site in native top-down MS of membrane proteins, however many other helices with a greater number of glycines (helix 9, 10, and 11, containing 6, 5, and 3 glycine residues, respectively) appear impervious to fragmentation by IRMPD. This observation implies that specific amino acid pairs are not good predictors of fragmentation propensity. Next, we mapped the relative abundance of the fragments onto the structure of the AmtB trimer. We observed that high intensity fragments typically do not originate from amino acids which face the lipid bilayer but instead are mostly in the core or at subunit interfaces (Fig. 4C top). This was intriguing, as most native top-down MS studies have observed bond cleavage primarily at solvent exposed regions in quaternary assemblies.^[14d]^

We attempted to rationalize why fragment ions would form in internal regions of the protein assembly by following changes to the AmtB assembly using an all-atom MD simulation at increasing temperatures of 300 K, 400 K and 500 K. After a 100 ns simulation at 300 K, we heated the protein to 400 K (for 100 ns), and then subjected the final structure to an additional 100 ns of high temperature (500 K) simulation. The structure remains relatively stable for the entire 300 ns simulation, as the protein exhibited limited (<10 nm) fluctuations, indicating the overall topology of the complex was maintained (Fig. S10). Inspecting snapshots of secondary structure across the trajectory, we find helices 5, 9, 10 and 11, which appear impervious to fragmentation, all lose the majority of their helical structure at elevated temperatures (Fig. 4C bottom and Fig. S11). By contrast, the α-helical character of helices 4, 6, and 8 is maintained in the regions that are readily fragmented. It has been demonstrated previously that helices *in vacuo* are stabilized when a charge-carrying amino acid “caps” the helix,^[32]^ forming electrostatic interactions with the dipoles of the peptide bonds comprising the helix.^[33]^ Indeed, at least one charge remote fragment is found originating from a nearby loop, adjacent to a fragmented helix. Thus, it would appear that successive cleavage of adjacent amino acids arises from charge-induced dissociation driven by mobile protons along regions where the α-helix is stabilized through this mechanism. However, there are still regions which retain localized structure but resist fragmentation; this can be reconciled given the extreme stabilization of α-helices in the gas phase,^[34]^ as well as differences in van der Waals interactions and hydrogen bonding networks which may contribute to individual stability, even at elevated temperatures. Still, such a peculiar fragmentation pattern suggests that residual influence from the original protein structure is reflected in the fragment ions observed.

To explore the extent that fragment ions can be used to recover details of the antecedent structure, we extended our investigation to include two class A GPCRs, the beta-1-adrenergic receptor (β_1_AR) and the adenosine A_2A_ receptor (A_2A_R). All class A GPCRs share a common architecture,^[35]^ and both receptors maintain near complete conservation of tertiary structure, but only share <35% sequence homology. Comparison of their fragmentation pathways would therefore allow us to separate structural variation from amino acid sequence effects. β_1_AR and A_2A_R consist of long N-terminal extracellular domains followed by seven transmembrane helices separated by alternating cytoplasmic and extracellular loops. We first optimized laser output power and irradiation time to liberate these protein ions from ionized detergent micelles (Figs. S12 and S13). The native mass spectra yield measured masses of both β_1_AR (40976 ± 0.5 Da) and A_2A_R (46527 ± 0.5 Da). These masses are lower than the anticipated sequence masses (40980 Da and 46535 Da) and are consistent with the formation of two and four disulfide bridges, respectively. The three most abundant charge states (16+, 17+ and 18+) of each GPCR were isolated and subjected to fragmentation via IRMPD using a laser output power of 7.8-8.4 W for 5 ms. Despite the lack of sequence homology (<35%), similar sequence coverage for β_1_AR (28 %) and A_2A_R (19%) was observed (Figs. S14 and S15).

**Figure 5:**
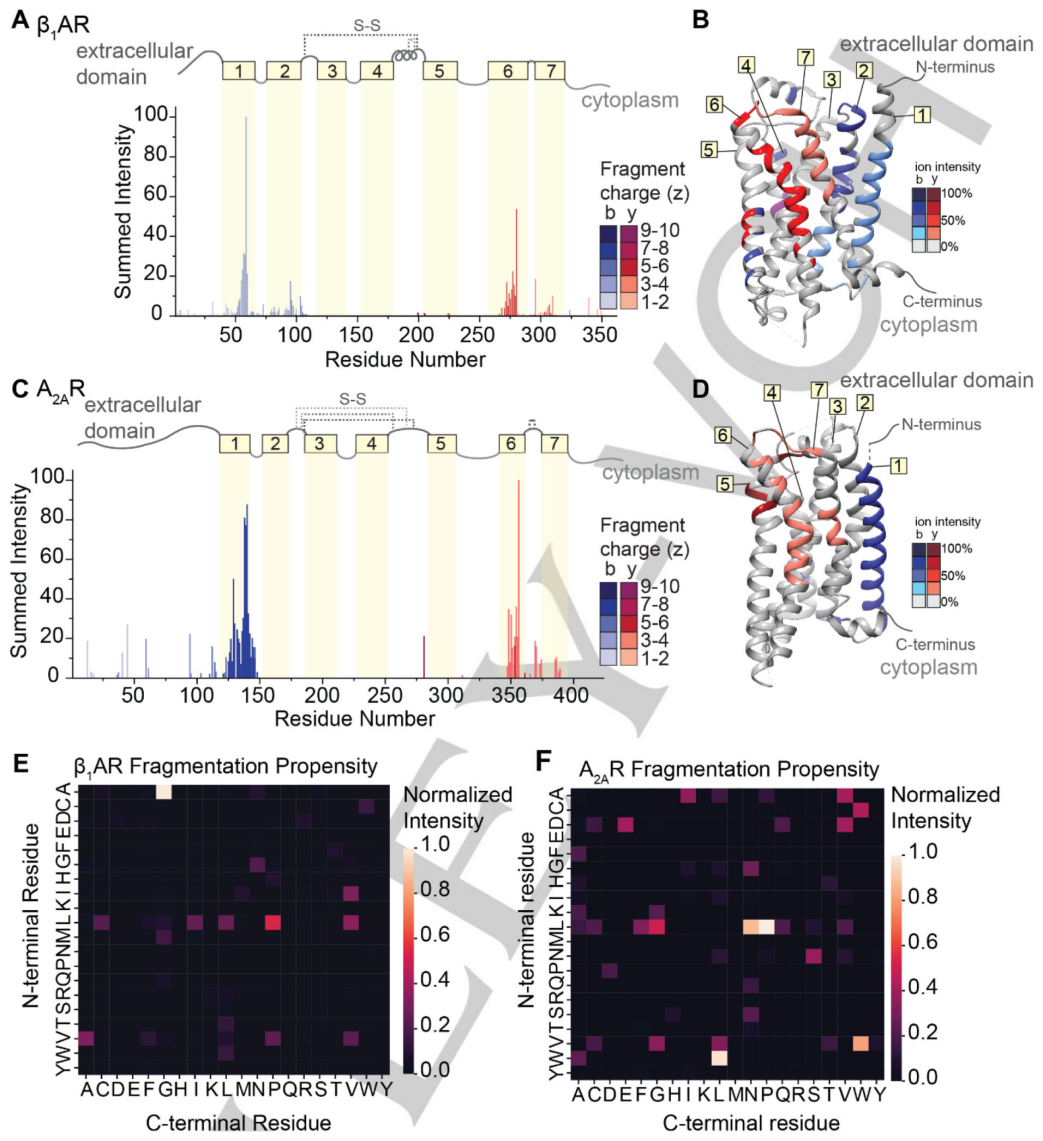
IRMPD of β_1_AR and A_2A_R. (A and C) Bar plots of fragment abundance relative to the origin of cleavage (residue number), where the abundance represents the sum of normalized intensities of fragments originating from each site. The color intensity of the bar represents the weighted average charge of each assigned fragment. The topological domains of each GPCR are overlaid onto the bar plot; α-helical transmembrane regions are represented by yellow boxes and numbered 1 through 7. The transmembrane regions are connected by extracellular and cytosolic loops, represented by gray lines. The extracellular loops joined by disulfide bonds are represented by gray dashed lines. (B and D) Sites of backbone cleavage are overlaid onto the structures of β_1_AR (PDB: 4GPO) and A_2A_R (PDB: 6GDG) using the same color scheme as in A and C. (E and F) Heat maps of fragmentation abundance relative to the assigned cleavage at amino acid pairs were generated using the normalized abundances of unique b-type and y-type fragments with respect to their origin in the protein sequence.

The normalized intensities of the fragments were then plotted against the assigned site of cleavage (Fig. 5A and C). To first explore connections between amino acid pair and fragmentation propensity, the normalized intensities of the 119 unique b- and y-type fragments for β_1_AR were used to generate a heat map of fragment intensity relative to cleavage at specific amino acid pairs. Again, we observed high propensity for fragmentation at A|G residue pairs followed by several preferential cleavages at L|X (Fig. 5E). The two most abundant fragments in β_1_AR originate from position 60 (A|G) and position 279 (L|P), with normalized intensities of 100% and 50%, respectively. For A_2A_R, the intensities and positions of the 72 unique b- and y-type fragment ions were used to generate a similar heat map (Fig. 5F). Apart from high propensity X|P bond cleavage, the two heatmaps are markedly different, indicating that there does not appear to be a preference for amino acid cleavage for these two receptors.

By contrast, we observe highly similar localization of fragmentation relative to key secondary structural domains. Considering first β_1_AR, we observe highly intense fragments corresponding to successive cleavage along the peptide backbone within helices 1, 2, 6, and 7, while only a few low abundance fragments are observed from the soluble extracellular and cytoplasmic domains (Fig. 5A). There is a marked absence of fragment ions originating from helices 3, 4, and 5, which can be attributed to the presence of two disulfide bonds. Turning to A_2A_R, we observe similar fragmentation patterns as above with highly abundant, successive fragmentation in helices 1 and 6, as well as some lower abundance fragments localized to helix 7 (Fig. 5C). The absence of fragments originating from the innermost helices (3 through 5) is again attributed to extensive disulfide bonding networks. When the sites of fragmentation are overlaid onto the structures of each GPCR, the similarities in secondary structure-specific fragmentation patterns are evident (Fig. 5B and D).

To compare to traditional collision-based approaches, we subjected the same isolated charge states of both GPCRs to fragmentation by IRMPD versus higher-energy collisional dissociation (HCD) in back-to-back experiments (Figs. S16 and S17). We note that, on this instrument, we found that the acceleration potential into the ion-routing multipole was too low to induce fragmentation of AmtB trimers by HCD. For β_1_AR, fragment ions generated by IRMPD were of higher average molecular weight of 8866 Da compared to 5843 Da for HCD. Consequently, the fragment ions produced by IRMPD also retained a higher average charge (4.7+ vs 3.6+ z). Ultimately, fragmentation by IRMPD resulted in higher sequence coverage of β_1_AR at 28% compared to 17% for HCD (Fig. S16). Similar trends were observed for A_2A_R, with 19% coverage by IRMPD compared to 9% for HCD (Fig. S17). Although there are qualitative differences in the fragment ions generated by IRMPD versus HCD for the GPCRs, it should be noted that the sites at which fragment ions originate are similar across both modalities. The framework for peptide and protein dissociation via mobile protons is well established,^[36]^ and IRMPD and HCD are both expected to fragment according to this mechanism.^[37]^ Improvements gained by IRMPD can be attributed to (i) enhanced migration of protons, afforded by the slow pooling of energy in IRMPD, and (ii) the paucity of charged side chains in membrane proteins compared to soluble proteins which allows protons to migrate further. Together these two factors result in larger and more highly charged fragment ions.

Again, we investigated the influence of structure on the fragmentation for both GPCRs using molecular dynamics simulations. At the highest temperature of 500 K, both GPCRs exhibit changes associated with the loss of initial structure and protein collapse *in vacuo* (Fig. S18). Interestingly, snapshots taken from the final structure at 500 K reveal regions in both GPCRs which retain α-helical character and correlate well with observed sites of cleavage (Fig. S19). These observations further support our proposal that structural features persist and enable fragmentation by IRMPD. Together these results highlight that stabilized transmembrane helices, likely derived from nearby protonation sites, render them prone to a higher frequency of backbone cleavages.

## Conclusion

Here, we showed that we can fully liberate membrane proteins from a range of detergent micelles with IR irradiation in the high pressure QLIT of an extensively modified Orbitrap Eclipse mass spectrometer. Then, by increasing our laser power, we obtain sequence-informative fragment ions released directly from membrane proteins and their complexes. By demonstrating that the removal of detergents by IR light is versatile and highly controllable, our advancement paves the way for preserving and identifying fragile non-covalent interactions between membrane proteins and ligands. This method also has great promise for liberating membrane proteins from mimetics that have been notoriously difficult to fully remove in the mass spectrometer such as lauryl maltose neopentyl glycol (LMNG), and glycoldiosgenin (GDN); both detergents are widely used in structural biology but not yet MS-compatible with current collision-based approaches. This paves the way for top-down analysis of a greater number of membrane proteins such as ion channels, transporters, and solute carriers which have unique solubilization profiles in different types of detergents.^[9]^

It is notable that the use of laser light to rid membrane proteins of detergent also affords a dramatic enhancement in MS^1^ signal intensity, which further enables top-down MS of native proteins. Rich fragmentation spectra were obtained for membrane proteins of different molecular weights, topologies, and oligomeric states. We have also made key observations about how natively folded integral membrane proteins of various sizes and topologies fragment. Our attempts to rationalize fragmentation propensity based on specific amino acid pairs alone yielded unsatisfying correlations. Notably, we observed a high propensity for the generation of sequential fragments (i.e., sequence tags) within transmembrane helices.

Helices are known to interact more closely with each other in membrane proteins than in soluble proteins.^[38]^ This proximity may contribute to the extensive transmembrane cleavage we observe within specific transmembrane helices. It is interesting to note that in the AmtB complex, high abundance fragments originate from amino acids mostly internal to the structure of the protein where helix-helix interactions are expected to dictate substrate translocation through the channel.^[39]^ Similarly, the high intensity fragments in β_1_AR and A_2A_R occur at similar α-helical regions despite sharing low sequence similarity.^[40]^ With these factors in mind, we need to consider the influence of structure of the ions immediately prior to dissociation to better explain the fragmentation patterns observed by IRMPD.

While extensive datasets on a greater number of membrane proteins will be required to fully rationalize these fragmentation patterns, these results imply that we can inform features of membrane protein topological organization. We found that sequence tags are almost exclusively obtained from cleavage localized to transmembrane regions. We highlight similarities to previous work using collisional dissociation of the seven transmembrane α-helical protein, bacteriorhodopsin, where fragmentation did not appear to be predictably correlated with transmembrane domains; some helices were accessible to dissociation and others were impenetrable.^[14a]^ It has been established that “capping” a helix with a charge-carrying basic amino acid dramatically enhances the structural stability of the helix *in vacuo*.^[38]^ Given that proton-directed fragmentation is the principal mechanism driving the peptide bond cleavage described herein, our results indicate that a nearby proton may stabilize the helix and also facilitate fragmentation in the α-helical transmembrane domains. We utilize MD simulations to rationalize this and found that some of the transmembrane regions which maintain their helicity at high temperatures are prone to fragmentation, giving credence to our hypothesis that protons can both stabilize and induce successive peptide bond cleavage through migration along the protein backbone, resulting in the fragmentation patterns observed above. The ability to extract structural information from native top-down MS of soluble and membrane proteins has been demonstrated using various activation modalities, so it is conceivable that an enduring influence of the protein ion structure is reflected in the distribution of product ions.^[14d, 17, 18e, 18f, 41]^

Altogether, membrane protein fragmentation remains challenging. Here, we present a framework for interpreting how fragment ions from α-helical membrane proteins arise. We find such fragment ions are preferentially generated from dissociation along successive residues in transmembrane helices. Most importantly, good (>20%) sequence coverage and sequence tags afforded by IRMPD allow for unambiguous protein ID. This is compulsory as the field of native mass spectrometry moves toward interrogating heterogeneous membrane proteins in context – that is, directly from their native membranous environments amid the cellular milieu.

## Supplementary Material

Supplementary material
