# Supplementary material for: Infrared Multiphoton Dissociation Enables Top-Down Characterization of Membrane Protein Complexes and G Protein-Coupled Receptors
Source: Angew Chem Int Ed Engl. Author manuscript; Available in PMC 2023 Oct 9. (PMC7615181; doi:10.1002/anie.202305694)
Supplement: Supplementary file 1 [file EMS188944-supplement-Supplementary_material.docx]

**Supporting Information** 

**Infrared Multiphoton Dissociation Enables Top-Down Characterization of Membrane Protein Complexes and G Protein-Coupled Receptors**

Corinne A. Lutomski,^1,2^ Tarick J. El-Baba,^1,2^ Joshua D. Hinkle,^3^ Idlir Liko,^4^ Jack L. Bennett,^ 1,2^ Neha V. Kalmankar,^1,2^ Andrew Dolan,^1,2^ Carla Kirschbaum,^5,6^ Kim Greis,^5,6^ Leonhard H. Urner,^1,2,7^ Parth Kapoor,^4^ Hsin-Yung Yen,^4,8^ Kevin Pagel,^5,6^ Christopher Mullen,^3^ John E. P. Syka,^3^ Carol V. Robinson^1,2^

^1^ Physical and Theoretical Chemistry Laboratory, Department of Chemistry, University of Oxford, UK

^2^ Kavli Institute for Nanoscience Discovery, Dorothy Crowfoot Hodgkin Building, University of Oxford, UK

^3^ Thermo Fisher Scientific, San Jose, CA, USA

^4^ OMass Therapeutics, Oxford, UK

^5^ Institute of Chemistry and Biochemistry, Freie Universität Berlin, Berlin, Germany

^6^ Fritz Haber Institute of the Max Planck Society, Berlin, Germany

^7^ Department of Chemistry and Chemical Biology, TU Dortmund University, Dortmund, Germany

^8^ Institute of Biological Chemistry, Academia Sinica, Taipei, Taiwan

Table of Contents

Experimental Methods	3

Figure S1. Liberation of AmtB from DDM detergent micelles	6

Figure S2. The effect of modulating laser output power and irradiation time for AmtB solubilized in DDM	7

Figure S3. Schematic of the instrument used for infrared action spectroscopy of detergent ions	8

Figure S4. Computed harmonic vibrations of protonated C_8_E_4_ headgroup	9

Figure S5. Solution-phase FTIR at increasing concentrations of DDM	10

Figure S6. Insets of fragment ion spectra with all annotated ions for the fragmentation of AmtB trimers via IRMPD	11

Figure S7. Graphical fragmentation map showing sequence coverage of AmtB.	12

Figure S8. Sites of repeat fragmentation in AmtB.	13

Figure S9. Fragmentation propensity based on amino acid pairs in AmtB.	14

Figure S10. Backbone root-mean square deviation (RMSD) and radius of gyration (Rg) of AmtB trimers in an all-atom gas phase molecular dynamics simulation.	15

Figure S11. Snapshots of AmtB secondary structure in the final frames of all-atom MD simulations.	16

Figure S12. The effect of modulating laser output power and irradiation time for β_1_AR solubilized in DDM detergent.	17

Figure S13. The effect of modulating laser output power and irradiation time for A_2A_R solubilized in DDM detergent.	18

Figure S14. Graphical fragmentation map showing sequence coverage of β_1_AR.	19

Figure S15. Graphical fragmentation map showing sequence coverage of A_2A_R.	20

Figure S16. Comparison of IRMPD and HCD for native top-down MS of β1AR	21

Figure S17. Comparison of IRMPD and HCD for native top-down MS of A_2A_R	22

Figure S18. Backbone root-mean square deviation (RMSD) and radius of gyration (Rg) of GPCRs, β_1_AR and A_2A_R, in all-atom molecular dynamics simulations.	23

Figure S19. Snapshots of GPCR secondary structure in the final frames of all-atom MD simulations	24

References	25

Experimental Methods

*Protein Expression and Purification*

*E. Coli* Ammonia channel (AmtB) was expressed and purified as described previously.[^e1] Briefly, AmtB was initially purified n 2× CMC DDM detergent by size exclusion chromatography using a 24 mL Superdex 200 Increase 10/300 column. Detergent exchange was carried out using a P6 biospin column (BioRad) into 200 mM ammonium acetate containing 2× CMC of C_8_E_4_, G1, or DDM detergent. 

Beta-1-adrenergic receptor (β_1_AR) and was expressed and purified in DDM as previously described.[^e2] Briefly, β_1_AR was overexpressed in Sf9 insect cells using a baculovirus expression system (Thermo Fisher). Protein was purified via affinity purification using a HiTrap TALON column (GE Healthcare) in buffer comprised of 20 mM Tris-HCl (pH 8.0), 350 mM NaCl, and 0.05% DDM. The protein was then buffer exchanged into 200 mM ammonium acetate containing 0.017% DDM immediately prior to MS analysis. 

The wide-type human Adenosine a2A receptor (A_2A_R) construct, fused to TrxA via a EAAAKA linker and modified with a N-terminal TEV protease cleavable 10×His tag, was expressed in Sf21 cells using a BacMam viral expression system. To improve protein mass homogeneity, a potential glycosylation site was mutated to alanine (N154A). Briefly, cells were seeded at 4million cells/mL and multiplicity of infection (MOI) of -0.5 was used. After 48 hours of expression, cells were harvested by centrifugation (1000 × g for 20 min at 4 °C) and resuspended in phosphate buffered saline (PBS). Cells were lysed by microfluidizer in 20mM Tris- HCl, pH 8, 1 mM EDTA and protease inhibitor. The cell debris were removed by centrifugation (5000 × g for 10 min at 4 °C) and membranes were pelleted by centrifugation at 180,000 × g, 4°C, for 2 hours. The membrane pellet was resuspended in 20 mM HEPES, pH 7.5, 1 mM EDTA and 1 mM phenylmethylsulfonyl fluoride (PMSF) and stored at -80 °C until use. A_2A_R was purified from membranes in extraction buffer consisting of 20 mM HEPES, pH 7.5, 300 mM NaCl, 1 mM EDTA, 1 mM PMSF, 100 µM NECA, protease inhibitor, 10 mM imidazole, and 2% DM and separated from the insoluble fraction by centrifugation 120,000 × g, 4°C, for 1 hour. The supernatant was filtered and diluted using resuspension buffer comprising 20 mM HEPES, pH 7.5, 300 mM NaCl, 1 mM EDTA, 1 mM PMSF, 100 µM NECA, 10 mM imidazole, and 0.15% DM prior to purification via immobilized metal affinity chromatography (IMAC). The protein was eluted in 20 mM HEPES, pH 7.5, 100 mM NaCl, 100 µM NECA, 300 mM imidazole, and 0.15% DM and buffer exchanged back into resuspension buffer. The 10×His tag was removed by TEV protease treatment followed by dialysis at 4 °C overnight in 10 mM HEPES, pH 7.5, 100 mM NaCl, 100 µM NECA, 20 mM imidazole, 10% glycerol and 0.15% DM. Following reverse IMAC, the protein was purified by size exclusion chromatography on a HiLoad 16/600 S75 column preincubated in 10 mM HEPES, pH 7.5, 100 mM NaCl, 100 µM NECA, 10% (v/v) glycerol and 0.002% LMNG or 0.02% DDM. The protein was concentrated to 1 mg/mL and stored at -80 °C. Prior to MS analysis, A_2A_R was buffer exchanged into 200 mM ammonium acetate containing 2× CMC DDM in small volume ZebaTM spin desalting columns (Thermo Fisher Scientific). 

*Native Mass Spectrometry*

Experiments were conducted on an Orbitrap Eclipse Tribrid mass spectrometer (Thermo Fisher Scientific) with two major modifications to improve performance on membrane protein complexes. First, a Synrad Firestar Ti60 CO_2_ continuous wave IR laser (10.6 um, 60W) was coupled to the instrument such that its beam was focused into the high-pressure cell of the dual cell quadrupole linear ion trap (q-LIT) and its timing and power output were controlled by the instrument software. This enabled both the use of IR irradiation for micelle removal as well as IRMPD of proteins and complexes for further dissociation. In addition, the operating frequency of the front-end transmission optics was lowered from ~2.4 to ~1.1 MHz. This served to improve the transmission of high m/z species by allowing for a greater trapping pseudopotential for higher m/z ions within the bounds of the Eclipse’s standard RF generator. Finally, the MP00 injection flatapole was extended to improve the collisional cooling of ions prior to traversing the MP0 curved bent flatapole ion guide. Proteins were ionized via static nanoelectrospray using an in-house-prepared gold-coated electrospray capillary held at ∼1.1 to 1.2 kV relative to the instrument orifice (heated to ∼100-200 °C). Ions were activated in the source using gentle activation energy (50 to 150 V source CID) before entering the next differential pressure region containing the bent flatapole. The instrument was operated in both standard and high-pressure modes (8 and 20 mtorr in the ion routing multipole, respectively). 

*Data Analysis and Software*

Fragments were assigned using TDValidator (Proteinaceous). Spectra were first annotated using a S/N cutoff of 7, max ppm tolerance of 20 ppm, sub ppm tolerance of 15 ppm, cluster tolerance of 0.35, minimum score of 0.6, charge states ranging from 1 to 10, using the distribution generator Mercury7. Assigned peaks were then manually inspected and incorrect assignments were removed.​ Internal fragments (peptides resulting from multiple fragmentation events that include neither the C-terminus nor the N-terminus of the protein) were not included in the analysis. Intensity information was exported to Origin Pro to generate bar plots summarizing fragmentation intensity relative to residue position. The fragmentation intensities were normalized by the charge state of the ion. The topological domains overlaid on the bar graphs were assigned according to those annotated in the Uniprot database. Disulfide bonds in β_1_AR and A_2a_R are well annotated in the database and were also included on the topological maps. 

	To examine the effect of primary structure on fragmentation propensity, the charge-normalized intensities for individual fragment ions were grouped based on the residues directly N-terminal and C-terminal to the fragmentation site. The summed intensity for each of the 400 possible residue combinations was then calculated and normalized to the most intense amino acid pair. Normalized intensities for each of the residue pairs were displayed as a heatmap using the Python 3.7 library Seaborn.

*IR Spectroscopy*

Gas-phase infrared (IR) action spectra of protonated detergents were measured on a custom-built instrument[^e3]^,^[^e4]. Briefly, the ionized detergents are captured in superfluid helium droplets, which constitute an IR-transparent, cryogenic spectroscopic matrix (0.37 K)[^e5]. Upon absorption of multiple IR photons, the intact ions are released from the droplets and detected by mass spectrometry. The detection of released ions as a function of the photon energy is an indirect measure for IR absorption. The detergents were dissolved in a 1:1 (v:v) water/methanol solution and diluted to a final concentration of 200 µM. For nano-electrospray ionization, needles were prepared in house from borosilicate capillaries pulled by a P-1000 micropipette puller (Sutter Instrument, USA) and coated with Pd/Pt by a sputter coater 108auto (Cressington, Germany). The detergents were ionized by applying needle voltages between 0.7–1.1 kV. Following ionization, the *m/z* of interest is selected in a quadrupole before the ions are deflected by a quadrupole bender into a hexapole ion trap. In the ion trap, the ions are thermalized by helium buffer gas and by additional cooling of the trap copper housing using liquid nitrogen (90 K). After thermalization, the buffer gas is pumped out. Superfluid helium droplets are generated by the expansion of pressurized helium (60 bar) through the cold nozzle (21 K) of a pulsed Even-Lavie valve (10 Hz repetition rate). The helium droplets traverse the ion trap coaxially and pick up trapped ions. Due to their high kinetic energy, the doped droplets overcome the trapping potential and travel towards the interaction region, where the pulsed beam of doped droplets overlaps with the pulsed beam of IR photons (10 Hz macropulse repetition rate) generated by the Fritz Haber Institute free-electron laser (FHI FEL)[^e6]. The released ions are detected on a time-of-flight mass analyzer and plotted as a function of the tunable wavenumber. The laser was scanned in steps of 2 cm^−1^ from 800 to 1700 cm^-1^, and each final spectrum was averaged from two individual scans.

Solution-phase infrared spectroscopy was carried out on a Varian Excalibur FTS 3500 FT-IR Spectrometer. Spectra were collected from 1% - 10% (w/v) detergent solutions dissolved in MilliQ water. Absorbance was measured between 600 - 4000 cm^-1^.

*Molecular Dynamics Simulations*

Simulations were run in vacuo using the OPLS-AA/L forcefield using GROMACS v2020.3, with GPU acceleration and without electrostatic or Lennard-Jones interactions cut-offs. The PDB structures were obtained from available crystal structures: 1U7G (Ammonia channel; AmtB), 4GPO (Beta-1 adrenergic receptor; β_1_AR) and 6GDG (Adenosine A2a receptor; A_2A_R). To automate the charge assignment step for gas phase simulations, ChargeRoulette script (https://github.com/andymlau/ChargeRoulette.git) was used. The structures were initially subjected to steepest-descent energy minimization, followed by 1 ns vacuum equilibration simulations, with randomly generated initial velocities. Subsequently, 100 ns production runs were conducted at temperatures 300K, 400K and 500K to reflect the progressive heating caused by IRMPD. VMD v1.9.3 was used to visualize the trajectories and produce molecular renderings.

*Density Functional Theory (DFT) Calculations*

For computation of harmonic vibrations of protonated C_8_E_4_, the molecule was truncated to feature the primary alcohol, two ethylene units and one ether oxygen (sum formula C_4_H_10_O_2_). The favored protonation site was determined using CREST[^e7] with the semiempirical method GFN2-xTB[^e8] and default settings. The most stable protomer was found to be protonated on the ether oxygen. The protonated molecule fragment was then subjected to a conformational sampling in CREST, and the most stable conformer was optimized at the PBE0+D3/6-311+G(d,p) level of theory in Gaussian 16.[^e9] The harmonic IR spectrum of the DFT-optimized structure was computed at the same level of theory and scaled by an empirical factor of 0.965, which has shown good agreement with experimental gas-phase IR spectra throughout different molecular classes (see for example references [^e10]^,^[^e11]^,^[^e12]). The results were visualized in GaussView 6.

Figure S1. Liberation of AmtB from DDM detergent micelles by IR irradiation reveals a well-resolved charge state series corresponding to the intact trimer with up to five bound phospholipids is observed. Similarly, lipids can be observed still bound to the monomeric protein as well as DDM detergent-bound peaks. The mass spectrum was obtained using a laser output power of 3.3 W for an irradiation time of 200 ms.  Phospholipids have been assigned previously as highly selective and highly stabilizing phosphatidylglycerols.[^e13]

Figure S2. The effect of modulating laser output power and irradiation time for AmtB solubilized in DDM detergent.  At low laser output power and short irradiation times, the peaks corresponding to AmtB trimer appear at low abundance due to signal dilution across many adduct peaks. With increasing irradiation time, peaks corresponding to lipid-bound trimers are observed as the protein is liberated from DDM detergent micelles. These peaks dominate the mass spectrum ~6000 m/z. At intermediate laser output powers from short to long irradiation times, the relative abundances of peaks corresponding to trimer and monomer can be modulated. Finally, at high laser output power and short irradiation times, peaks corresponding to monomer and trimer are approximately equal in abundance, while at long irradiation times, the peaks corresponding to trimer are absent as the protein complex has expectedly dissociated into monomeric subunits (peaks <5000 m/z).

Figure S3. Schematic of the instrument used for infrared action spectroscopy of detergent ions in superfluid helium droplets. The ionized detergents are captured in superfluid helium droplets, which constitute an IR-transparent, cryogenic spectroscopic matrix (0.37 K) Upon absorption of multiple IR photons, the intact ions are released from the droplets and detected by time-of-flight mass spectrometry. The detection of released ions as a function of the wave number is an indirect measure for IR absorption.

Figure S4. Computed harmonic vibrations of protonated C_8_E_4_ headgroup. (A) shows the harmonic IR spectrum of the DFT-optimized structure computed at the PBE0+D3/6-311+G(d,p) level of theory and scaled by 0.965. Inset shows the simplified structure of the C_8_E_4_ headgroup protonated at the ether oxygen. 21 vibrational bands are observed in the calculated spectrum, including a band at 945 cm-1. The vibrations giving rise to the harmonic mode at 945 cm^-1^ are provided as a .gif and provided as supplementary material (Movie S1). Two arbitrary frames from Movie S1 (B and C) are shown to demonstrate the coupled contributions from O-H bending, C-C stretching, C-H bending, and C-O stretching. 

Figure S5. Solution-phase FTIR at increasing concentrations of DDM. (A) shows absolute absorbance values for solutions of DDM detergent at concentrations of 0.5% w/v, 1% w/v, and 10% w/v DDM and (B) shows the normalized intensities from the spectra shown in (A). Despite measuring FTIR at concentrations higher than those used in a native mass spectrometry experiment (e.g. 0.017% w/v for DDM) the spectral features remain unchanged across the detergent concentrations tested. 

Figure S6. Insets of fragment ion spectra with all annotated ions for the fragmentation of AmtB trimers via IRMPD. Peaks highlighted by red triangles represent assigned fragment ions that have a signal-to-noise ratio >10. The b- and y-type assignments are annotated above each peak. 

Figure S7. Graphical fragmentation map showing sequence coverage of AmtB. Fragments observed after IRMPD by irradiating the ions with a laser output power of 9 W for 5 ms following ion trap isolation of the 19+ charge state of the trimeric complex at m/z 6674. Fragments are assigned to cleavages along the protein backbone, where 106 residues out of a total of 406 residues were cleaved resulting in 26% sequence coverage. 

 

Figure S8. Sites of repeat fragmentation in AmtB. Plot shows a histogram displaying the number of unique charge states of fragment ions that have been assigned to amino acid cleavages along the protein backbone (residue number). The blue bars represent b-type ions, and the red bars represent y-type ions.

Figure S9. Fragmentation propensity based on amino acid pairs in AmtB. Heat map of the abundance of assigned fragments relative to their corresponding cleavage at specific amino acid pairs. Plot was generated using the normalized abundances of 106 unique b- and y-type fragments.

Figure S10. Backbone root-mean square deviation (RMSD) and radius of gyration (Rg) of AmtB trimers in an all-atom gas phase molecular dynamics simulation. After a 100 ns equilibration at 300 K, the protein was heated to 400 K (for 100 ns), and the final frame again was subjected to an additional 100 ns of high temperature (500 K) equilibration.

Figure S11. Snapshots of AmtB secondary structure in the final frames of all-atom MD simulations. Simulations were equilibrated for 100 ns at each temperature, where the final frame of the previous temperature was used to initiate the subsequent 100 ns equilibration.  Areas shaded in blue represent b-type ions and areas in red represent y-type ions, the color intensity corresponds to the abundance of the fragment(s) assigned to each cleavage site. Abundances reflect those in the bar plot shown in main text Figure 4A. 

Figure S12. The effect of modulating laser output power and irradiation time for β_1_AR solubilized in DDM detergent.  At low laser output power and short irradiation times, the peaks corresponding to β_1_AR share near equal intensity with peaks corresponding to DDM clusters at m/z <2000.  With increasing irradiation time, peaks corresponding to β_1_AR (m/z 2000 to 4000) appear more abundant as the protein is effectively liberated from DDM detergent micelles. At intermediate laser output power (4.8 W) and 25 ms irradiation, peaks corresponding to β_1_AR are the most abundant features of the mass spectrum.

Figure S13. The effect of modulating laser output power and irradiation time for A_2A_R solubilized in DDM detergent.  At low laser output power and short irradiation times, the peaks corresponding to A_2A_R are well resolved with few adduct peaks corresponding to residual LMNG detergent (introduced during protein purification). At mid-range laser output power of 4.8 W and 25-50 ms irradiation times, peaks corresponding to A_2A_R dominate the spectrum between m/z 3000 and 5000.

Figure S14. Graphical fragmentation map showing sequence coverage of β_1_AR. Map shows the assigned fragments generated by individual dissociation via IRMPD for three isolated charge states (+18, +17, and +16 at 2277, 2411, and 2561 m/z, respectively). The isolated ions were irradiated with 7.2-8.4 W laser output power for 5 ms. Fragments assigned correspond to cleavages at 102 residues out of a total of 362 resulting in 28% sequence coverage.

Figure S15. Graphical fragmentation map showing sequence coverage of A_2A_R. Map shows the assigned fragments generated by individual dissociation via IRMPD for three isolated charge states (+18, +17, and +16 at 2586, 2737, and 2909 m/z, respectively). The isolated ions were irradiated with 10.8-12.0 W laser output power for 5 ms. Fragments assigned correspond to cleavages at 81 residues out of a total of 424 resulting in 19% sequence coverage.

Figure S16. Comparison of IRMPD and HCD for native top-down MS of β1AR. (A and B) representative MS^2^ spectra for the isolated 17+ charge state fragmented by IRMPD (A) and HCD (B). (C and D)  Plots of fragment abundance relative to the origin of cleavage (residue number), where the abundance represents the sum of normalized intensities of fragments originating from each site in IRMPD (C) and HCD (D). The color intensity of the bar represents the weighted average charge of each assigned fragment. The topological domains of are overlaid onto the bar plot; α-helical transmembrane regions are represented by yellow boxes and numbered 1 through 7. (E and F) Histogram displaying the number of unique charge states of fragment ions that have been assigned to amino acid cleavages along the protein backbone (residue number) for IRMPD (E) and HCD (F). (G and H) Graphical fragmentation maps showing sequence coverage obtained by IRMPD (G) and (HCD) H.

Figure S17. Comparison of IRMPD and HCD for native top-down MS of A_2A_R. (A and B) representative MS^2^ spectra for the isolated 17+ charge state fragmented by IRMPD (A) and HCD (B). (C and D)  Plots of fragment abundance relative to the origin of cleavage (residue number), where the abundance represents the sum of normalized intensities of fragments originating from each site in IRMPD (C) and HCD (D). The color intensity of the bar represents the weighted average charge of each assigned fragment. The topological domains of are overlaid onto the bar plot; α-helical transmembrane regions are represented by yellow boxes and numbered 1 through 7. (E and F) Histogram displaying the number of unique charge states of fragment ions that have been assigned to amino acid cleavages along the protein backbone (residue number) for IRMPD (E) and HCD (F). (G and H) Graphical fragmentation maps showing sequence coverage obtained by IRMPD (G) and (HCD) H.

Figure S18. Backbone root-mean square deviation (RMSD) and radius of gyration (Rg) of GPCRs, β_1_AR and A_2A_R, in all-atom molecular dynamics simulations. After a 100 ns equilibration at 300 K, the proteins were heated to 400 K (for 100 ns), and the final frames again were subjected to an additional 100 ns of high temperature (500 K) equilibration.

Figure S19. Snapshots of GPCR secondary structure in the final frames of all-atom MD simulations. Simulations were equilibrated for 100 ns at each temperature, where the final frame of the previous temperature was used to initiate the subsequent 100 ns equilibration.  Areas shaded in blue represent b-type ions and areas in red represent y-type ions, the color intensity corresponds to the abundance of the fragment(s) assigned to each cleavage site. Abundances reflect those in the bar plots shown in main text Figure 5.

References

[^e1]: Reading, E. et al. The role of detergent micelle in preserving the structure of membrane proteins in the gas phase. Angew. Chem. Int. Ed. 2015, 54, 4577-4581.
[^e2]: Yen, HY., Liko, I., Song, W. et al. Mass spectrometry captures biased signalling and allosteric modulation of a G-protein-coupled receptor. Nat. Chem. 2022, 14, 1375–1382.
[^e3]: González Flórez, A. I.; Mucha, E.; Ahn, D. S.; Gewinner, S.; Schöllkopf, W.; Pagel, K.; von Helden, G. Charge-Induced Unzipping of Isolated Proteins to a Defined Secondary Structure. Angew. Chem. Int. Ed. 2016, 55, 3295-3299.
[^e4]: Mucha, E.; González Flórez, A. I.; Marianski, M.; Thomas, D. A.; Hoffmann, W.; Struwe, W. B.; Hahm, H. S.; Gewinner, S.; Schöllkopf, W.; Seeberger, P. H.; et al. Glycan Fingerprinting via Cold-Ion Infrared Spectroscopy. Angew. Chem. Int. Ed. 2017, 56, 11248-11251.
[^e5]: Toennies, J. P.; Vilesov, A. F. Superfluid Helium Droplets: A Uniquely Cold Nanomatrix for Molecules and Molecular Complexes. Angew. Chem. Int. Ed. 2004, 43, 2622-2648.
[^e6]: Schöllkopf, W.; Gewinner, S.; Junkes, H.; Paarmann, A.; von Helden, G.; Bluem, H.; Todd, A. M. M. The new IR and THz FEL Facility at the Fritz Haber Institute in Berlin. Proc SPIE 2015, 9512, 95121L.
[^e7]: Pracht, P.; Bohle, F.; Grimme, S. Automated exploration of the low-energy chemical space with fast quantum chemical methods. Phys. Chem. Chem. Phys. 2020, 22, 7169-7192.
[^e8]: Bannwarth, C.; Ehlert, S.; Grimme, S. GFN2-xTB-An Accurate and Broadly Parametrized Self-Consistent Tight-Binding Quantum Chemical Method with Multipole Electrostatics and Density-Dependent Dispersion Contributions. J. Chem. Theory Comput. 2019, 15, 1652-1671.
[^e9]: Gaussian 16, Rev. A.03; 2016.
[^e10]: Mucha, E.; Marianski, M.; Xu, F.-F.; Thomas, D. A.; Meijer, G.; von Helden, G.; Seeberger, P. H.; Pagel, K. Unravelling the structure of glycosyl cations via cold-ion infrared spectroscopy. Nat. Commun. 2018, 9, 4174.
[^e11]: Kirschbaum, C.; Greis, K.; Polewski, L.; Gewinner, S.; Schöllkopf, W.; Meijer, G.; von Helden, G.; Pagel, K. Unveiling Glycerolipid Fragmentation by Cryogenic Infrared Spectroscopy. J. Am. Chem. Soc. 2021, 143, 14827-14834.
[^e12]: Thomas, D. A.; Chang, R.; Mucha, E.; Lettow, M.; Greis, K.; Gewinner, S.; Schöllkopf, W.; Meijer, G.; von Helden, G. Probing the conformational landscape and thermochemistry of DNA dinucleotide anions via helium nanodroplet infrared action spectroscopy. Phys. Chem. Chem. Phys. 2020, 22, 18400-18413.
[^e13]: Laganowsky, A.; Reading, E.; Allison, T.M.; Ulmschneider, M. B.; Degiacomi, M. T.; Baldwin, A. J.; Robinson, C. V. Membrane proteins bind lipids selectively to modulate their structure and function. Nature. 2014, 510, 172-175.
